# Adapting the Girandole framework for cross-disciplinary social justice reflection in health professions education

**DOI:** 10.3389/fmed.2026.1942107

**Published:** 2026-10-09

**Authors:** Enrico De Luca, Bilkis Islam-Ali, Yusra Siddiqui

**Affiliations:** 1 Department of Nursing and Midwifery, College of Medicine and Health, University of Birmingham, Birmingham, United Kingdom; 2 Department of Pharmacy, College of Medicine and Health, University of Birmingham, Birmingham, United Kingdom; 3 Department of Biomedical Sciences, College of Medicine and Health, University of Birmingham, Birmingham, United Kingdom

**Keywords:** equality, diversity and inclusion, girandole framework, health professions education, hidden curriculum, interprofessional education, reflective practice, social justice

## Abstract

Equality, diversity and inclusion (EDI) are increasingly embedded within the standards and strategic priorities of health professions education. However, persistent inequities, hidden curricula and differences between institutional commitments and learners' lived experiences indicate a need for approaches that move beyond policy compliance towards critical, socially informed reflection. This Perspective paper explores the cross-disciplinary adaptation of the Girandole Framework, originally developed in nursing education, for application across health professions education. The framework uses six interconnected domains—Power, Hidden Curriculum, Professional Values, Personal Values, Culture, and EDI Education and Awareness—to support examination of the individual, cultural and structural influences shaping educational and professional experiences. An exploratory cross-disciplinary workshop involving healthcare professional students informed the adaptation of the framework. Informal discussions suggested that Power, Culture, and Personal and Professional Values were readily connected to learners' experiences. In contrast, Hidden Curriculum and EDI Education and Awareness required clearer language, practical examples and additional educational scaffolding. These insights informed the refinement of the framework's reflective prompts and the development of examples relevant to nursing, pharmacy, biomedical science and transferrable to other health professions. The adapted Girandole offers a shared language through which students and educators can critically examine power relationships, informal norms, professional cultures and educational omissions. Potential future applications include clinical and laboratory placements, professionalism teaching, portfolio-based learning, interprofessional education and curriculum review; however, these applications require empirical evaluation. The framework is intended to complement established reflective models by foregrounding equity and social justice. Further student–educator co-production and empirical evaluation are needed to assess its contribution to EDI literacy, belonging, reflective practice and meaningful organisational change across health professions curricula.

## 1 Introduction

Equality, diversity and inclusion (EDI) have become central priorities within health professions education, reflecting the need to prepare graduates who can deliver equitable, culturally responsive, and socially just care. Despite increasing emphasis on inclusive curricula and learning environments, challenges relating to belonging, representation, differential attainment, power dynamics, and hidden curricula persist across healthcare disciplines. Addressing these issues requires approaches that move beyond policy compliance and support critical reflection on how equity and social justice are experienced in educational and clinical contexts.

This paper emerged from a collaboration between academics from Nursing, Pharmacy, and Biomedical Sciences at the University of Birmingham who identified shared concerns relating to EDI. Building on the Girandole Framework (1), originally developed within nursing education as an EDI-focused reflective tool, we explore its adaptation for use across health professions education. This paper describes the Girandole cross-disciplinary adaptation and ongoing refinement, highlighting its potential to support meaningful reflection on social justice across healthcare curricula and informing future research, student co-production, and EDI-focused educational initiatives.

### 1.1 Social justice, EDI and the need for cross-disciplinary reflection

Healthcare professionals work within increasingly diverse societies and must be able to provide equitable, person-centred care to patients from a wide range of cultural, social and ethnic backgrounds. Consequently, health professions education has a responsibility not only to promote EDI, but also to prepare students to recognise and respond to the structural inequalities that contribute to health disparities (2).

Across the United Kingdom, EDI is firmly embedded within the expectations of professional regulators. Standards issued by the Nursing and Midwifery Council (NMC), General Pharmaceutical Council (GPhC) and General Medical Council (3, 4) emphasise inclusive learning environments, the reduction of discrimination, and the promotion of fairness throughout education and professional practice (4–6). However, despite these commitments, inequities continue to be reported across healthcare education and the wider workforce. Differential attainment, ethnicity-related awarding gaps, experiences of discrimination, and unequal opportunities for progression remain persistent concerns across several health professions (7, 8).

This mismatch between institutional aspirations and lived experience suggests that embedding EDI within healthcare education requires more than policy statements or isolated teaching sessions. Rather than being presented as a discrete curricular topic, EDI should provide a lens through which all aspects of healthcare education and practice are critically examined. A social justice approach therefore encourages students and educators to engage with broader questions of power, representation, belonging, privilege and structural inequality, while recognising the influence of the hidden curriculum. These often shape professional identity, learners' sense of belonging and understandings of inclusive practice as powerfully as formal teaching. Addressing these issues requires structured reflective approaches that help learners critically examine these interconnected dimensions of educational and professional life.

Although healthcare disciplines differ in their professional contexts, they share many of the same social justice challenges. Consequently, there is value in adopting reflective approaches that provide an adaptable language for examining these issues across disciplinary boundaries. Such approaches can support dialogue between educators and students while encouraging critical engagement with the social, cultural and structural factors that shape healthcare.

Reflective approaches can provide tools for examining inclusion across disciplinary boundaries, facilitating dialogue and supporting critical exploration of strengths and gaps within curricula. Reflection is a cornerstone of professional development in health professions education, but meaningful engagement with social justice requires structured frameworks that help learners examine power dynamics, organisational culture, professional values, representation, and lived experience. Without such scaffolding, educators may struggle to translate EDI ambitions into practice, while students may lack the tools to recognise and challenge inequity. The need, therefore, is not simply for more EDI content but for practical pedagogical approaches that support critical engagement with the social, cultural, and structural factors shaping healthcare education and practice. It is within this context that the current project explores the adaptation of the Girandole Framework as a cross-disciplinary reflective tool for social justice learning.

### 1.2 Existing reflective EDI frameworks

Higher education institutions in the UK operate within a well-established EDI landscape shaped by legislation, institutional strategies, and national accreditation schemes such as Athena SWAN and the Race Equality Charter (9). These initiatives have played an important role in promoting organisational accountability and addressing inequalities relating to gender, race, disability, and other protected characteristics. Alongside these structural approaches, universities have increasingly adopted concepts such as intersectionality, decolonisation of the curriculum, and inclusive educational practices to address more complex and interconnected forms of inequality.

Within health professions education, reflective practice has long been recognised as a cornerstone of professional development. Frameworks such as Gibbs' Reflective Cycle (10), Schön's reflective practice model (11), Johns' Model of Structured Reflection (12), and Mezirow's transformative learning theory (13) are widely used to support students in examining their experiences, assumptions, and professional behaviours. Gibbs' cyclical stages, Schön's distinction between reflection-in- and reflection-on-action, and Johns' cue-based questioning across aesthetic, ethical and empirical ways of knowing each provide valuable structures for reflection, but none were specifically designed to explore issues of equity, diversity, inclusion, power, or structural inequality. Their cues prompt learners to examine what happened and how they responded, rather than whose voice was privileged or what structural conditions shaped the encounter.

In response to this limitation, EDI-specific approaches have increasingly emerged within healthcare education. Cultural competence has traditionally been the dominant framework, encouraging healthcare professionals to develop knowledge and skills for working with diverse populations. More recently, cultural humility has shifted the focus towards lifelong self-reflection, critical awareness of personal biases, and recognition of power differentials within healthcare encounters (14). Related concepts such as racial humility and cultural competency have further expanded these discussions by emphasising structural inequities and socially responsive practice (15).

Although EDI is increasingly embedded within higher education curricula and professional programmes, translating these commitments into practice remains challenging. Educators and students often lack tools to examine how power, inclusion, exclusion, hidden curricula, and organisational culture shape learning experiences. While reflective models focus on process and EDI frameworks often emphasise knowledge or organisational change, few combine these dimensions. The Girandole Framework addresses this gap by providing a structured lens for exploring social justice in health professions education.

## 2 The Girandole framework

The focus of this paper is the Girandole Framework^1^ adaptation and early implementation within a new cross-disciplinary context. The Girandole's domains/vanes collectively shape the EDI culture in clinical learning environments (Figure 1).

**Figure 1:**
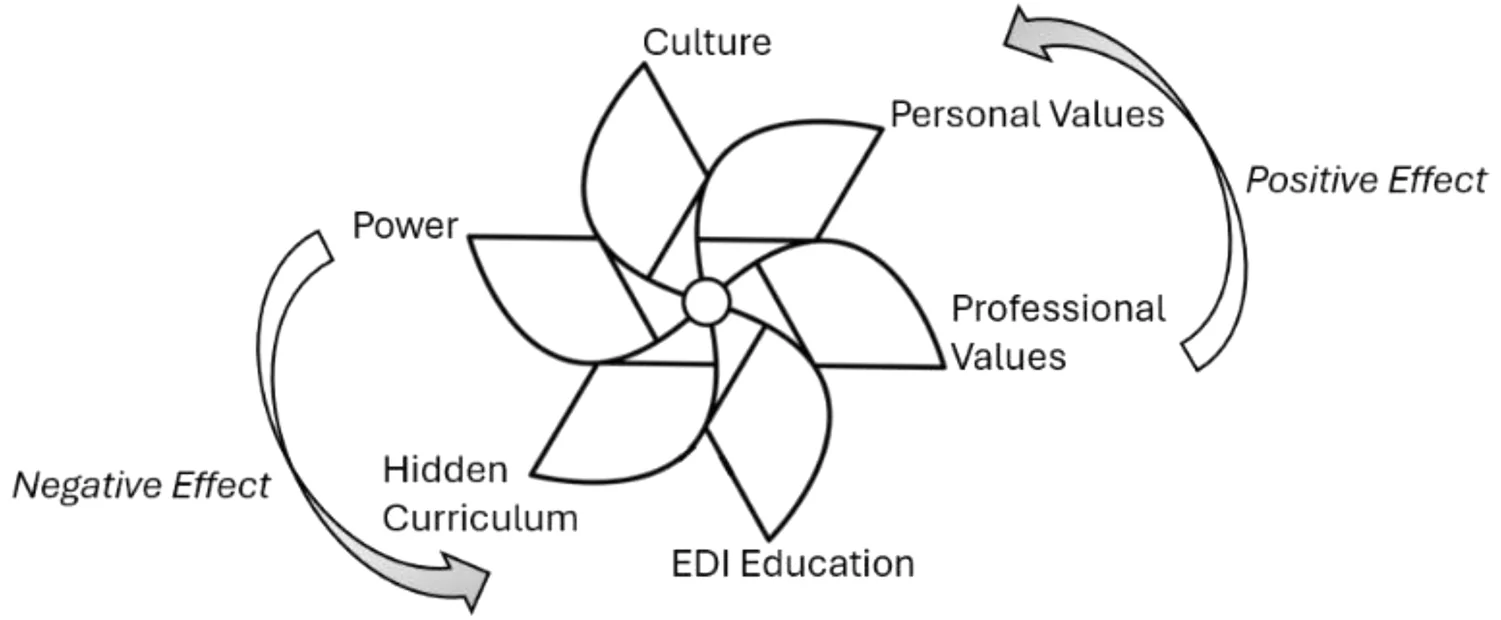
The Girandole framework (1).

The framework uses the metaphor of a spinning pinwheel to represent the dynamic and interdependent nature of these forces: depending on whether each vane operates as an enabler or a barrier, the Girandole can rotate in a positive or negative direction. Applied to real-world scenarios and vignettes, the framework prompts students, educators, and institutions to examine not only what happens in clinical and educational settings but how and why (Table 1).

**Table 1:** Structure of the Girandole framework.

Vane	Definition
Power	Power in learning environments operates through both formal roles and informal social dynamics, shaping behaviour and decisions, and while its constructive use can challenge discrimination and promote inclusion. Unchecked power may marginalise vulnerable groups and normalise exclusion, making it essential to reflect on how confidence, communication styles and unspoken hierarchies influence interpersonal dynamics.
Hidden Curriculum	Elements/contents in communication, behaviour, teaching, (unspoken) rules of practice that carry subliminal messages, convey institutional slangs and values that affects learning and subsequently care. Identifying and addressing elements of the hidden curriculum can be challenging; however, it represents a valuable opportunity for critical reflection and professional learning for both students and practitioners.
Professional Values	Professional values relate to the ethical principles and standards that guide behaviour and decision-making across medical and healthcare professions, as defined within discipline-specific codes of practice. Reflecting on how these values are enacted in real-world scenarios enables learners to identify ethical tensions and engage in critical reflection on accountability, role modelling and inclusive practice.
Personal Values	Individual beliefs and biases shape personal, education and clinical interactions by influencing assumptions and emotional responses, and through reflective alignment with professional ethics—supported by humility, self-awareness and a willingness to challenge one's own thinking—learners can strengthen ethical decision-making, navigate EDI issues more confidently and help create psychologically safe, inclusive environments.
Culture	Culture encompasses the shared norms, practices, attitudes and local traditions that shape workplace behaviour, and because it is dynamic and co-created—spanning ethnic, national, micro-cultural and generational influences— it can either foster belonging and equitable treatment or normalise discrimination and silence, making cultural understanding essential for identifying opportunities for positive change and advancing equity and inclusion.
EDI education and awareness	EDI education and awareness should be embedded across academic and clinical settings to ensure alignment between teaching and practice. Meaningful EDI learning is reflective and transformative, whereas tokenistic or inconsistent approaches undermine understanding and may perpetuate inequities, highlighting the need for sustained and intentional development to support continuous growth and equity awareness.

The Girandole vanes are interconnected, and each scenario may demonstrate overlapping elements, as they can represent different layers or nuances of the same topic.

### 2.1 Strengths and distinctive features

Based on this early cross-disciplinary adaptation, the Girandole appears to offer several distinctive potential strengths. First, it is grounded in lived experience and developed collaboratively by an interdisciplinary, multicultural team drawing on their own professional and personal encounters with EDI challenges, adapting real stories from students' clinical placements accounts and giving it an authenticity that top-down policy frameworks often lack. Second, it moves beyond individual-level cultural competence approaches by incorporating structural and systemic dimensions making it one of the few EDI reflective tools in nursing education to engage with structural competency. Third, its visual simplicity and metaphorical clarity make it accessible and memorable for students and educators across different contexts, supporting its practical integration into case-based learning, mentoring, and institutional review. Fourth, the framework is explicitly dynamic by acknowledging that the direction and intensity of each domain can shift depending on context, and that EDI work is a continuous process rather than a one-off intervention. Finally, the Girandole was designed to be transferable beyond nursing to other health professions and educational settings, and adaptable with its vanes, capable of being modified to suit different institutional or professional contexts.

## 3 Cross-disciplinary and student-informed adaptation

### 3.1 Exploratory implementation

To inform the cross-disciplinary adaptation of the Girandole Framework, the framework was introduced during an exploratory EDI workshop involving healthcare professional students. The workshop formed part of an educational development activity intended to stimulate discussion around social justice and EDI concepts and to support ongoing refinement of the framework. It was not designed as an empirical research study, and no systematic data collection or formal qualitative analysis was undertaken Rather, this paper reports the team's experience of the workshop and the wider process of adapting the framework across disciplines.

The student discussions elicited during the workshop served as an exploratory implementation rather than a formal evaluation, providing insights into both the strengths of the framework and areas for refinement. The discussions highlighted much about the current state of EDI in health professions education as it did about the framework itself.

During the presentation, it became immediately apparent that four of the six Girandole vanes — Power, Culture, Personal Values and Professional Values — resonated strongly and spontaneously with the student group. Students engaged quickly and confidently with these concepts, readily offering examples drawn from their own clinical placement and educational experiences. Discussions around power dynamics on workplaces, the cultural dimensions of patient care, and the tension between personal beliefs and professional conduct emerged organically and with considerable depth. Students were not simply understanding these concepts abstractly; they were recognising them as descriptions of situations they had already lived.

However, two vanes — Hidden Curriculum and EDI Education and Awareness — did not achieve the same level of engagement. Students appeared uncertain, disengaged or simply unable to connect these concepts to their experience. The research team post presentation, reflected on this pattern revealed two distinct but related explanations. First, the language and framing used to introduce these vanes remained too abstract and academic — intelligible perhaps to an educator familiar with the literature but insufficiently grounded in the everyday realities of student educational and placement experiences. Second, and more strikingly, student's recollection of any formal EDI training prior to this session was missing and so lacked the critical engagement with these concepts. They were being asked to evaluate and reflect on aspects of EDI that although potentially encountered within the curriculum, had not been sufficiently not internalised or translated into their understanding of practice. an important question for health professions education: how effectively are existing approaches to EDI enabling students to develop the knowledge, language and critical awareness needed to recognise and interrogate issues of power, hidden curricula and structural inequity? The observation echoes wider concerns raised in the literature, where EDI has been described as inconsistently embedded in healthcare professions universities curricula, often remaining tokenistic or superficial rather than transformative (16, 17). These discussions prompted consideration of.

This reflection prompted a reconsideration of how these two vanes are introduced and supported in practice. Therefore, the interprofessional groups worked on two questions and reflected on what and how the Girandole next move can be pathed.

### 3.2 Engaging with the hidden curriculum

The hidden curriculum refers to what students learn in clinical placements and educational settings not through formal teaching, but through the unspoken norms, values, hierarchies and behaviours absorbed through observation and social pressure (18). In EDI terms, it represents the gap between what an institution formally states it values — in its policies, charter mark awards and mandatory training — and what is modelled and rewarded in daily practice (19). Students recognise the hidden curriculum most readily through their own lived experiences, once given the language to name it. It is present in the ward where no one explicitly prohibits a student from addressing the consultant directly, yet the social consequences make the unwritten rule unmistakable within days. It operates where certain patients — disproportionately from minority ethnic backgrounds or with mental health conditions — wait longer for care, yet this pattern is never discussed or challenged (20).

### 3.3 The paradox of evaluating what you have not been taught

The EDI Education and Awareness vane asks students to reflect on how meaningfully EDI has been embedded in their curriculum and clinical placement, and to identify gaps between stated institutional commitments and lived educational experience. This is among the most important reflective tasks the Girandole offers — and, as the presentation experience described above revealed, among the most challenging for students to engage with. The difficulty is structural rather than intellectual: it is extraordinarily difficult to evaluate the quality or completeness of an education you have not yet received. This paradox is not merely pedagogical — it is political. Before students can meaningfully apply this vane, they require a brief but substantive introduction to what EDI means in clinical and educational practice. In practice, EDI in a clinical setting might look like: a ward team that routinely considers cultural, religious and linguistic factors as part of care planning; a mentor who openly acknowledges and addresses a student's experience of microaggression; a debrief session that explicitly examines how a patient's ethnicity or disability status shaped the care they received. Its absence might look like: EDI training that is delivered once and never revisited in practice; a placement culture where discriminatory language is normalised or laughed off; a curriculum in which the perspectives, histories and experiences of marginalised groups are invisible; or a student who feels unable to raise a concern about unfair treatment for fear of jeopardising their placement assessment.

These aspects suggest that, even where EDI content is present, students may still struggle to translate theoretical knowledge into critical awareness of how power, representation, and inclusion operate within professional and educational settings. In this context, the Girandole may provide a useful reflective bridge between institutional EDI initiatives and students' lived learning experiences.

The central argument of the EDI Education and Awareness vane is that without meaningful, integrated EDI education — not a single mandatory online module, but sustained, reflective, practice-embedded learning — students will arrive in clinical and educational environments carrying unexamined biases they do not know they have, and without the tools to recognise or address them.

### 3.4 From reflection to application

These insights informed the next stage of framework development. Rather than viewing the difficulties encountered with the Hidden Curriculum and EDI Education and Awareness vanes as weaknesses, the project team treated them as opportunities to refine the framework and strengthen its pedagogical accessibility. The revised prompts illustrate how the Girandole could be operationalised beyond its original context. By translating its domains into practical reflective questions and context-specific examples, the framework has the potential to support teaching, learning and professional development across different health professions. These proposed applications require evaluation in authentic educational settings. Table 2 presents the adapted reflective prompts together with examples of how they might be applied across healthcare education contexts.

**Table 2:** Reflective prompts and examples to guide Girandole framework applications.

Vane	Prompts for reflective practice	Elements to notice in practice and education	Examples across the healthcare education context
Power	1. Who holds official power positions and who does not? 2. Who has control or direct influence? 3. Did anyone feel unable to speak up? 4. Is there an unofficial power element?	Hierarchy, voice, inclusion/exclusion.	A senior clinician dismisses a junior's concern, and no one challenges it. A student feels unable to question a mentor's decision. A student questioning an academic during teaching but over-ruled/stood down or deliberately embarrassed; student embarrassed in class for late attendance but has genuine reason due to personal circumstances or similar.
Hidden Curriculum	1. What did I learn from what people did (not what was taught)? 2. What behaviour seemed acceptable here? 3. Was I encouraged or discouraged from asking questions?	Unspoken rules, silence, informal learning.	A student is told to ignore university teaching in clinical skills and “do it the ward way,” without explanation. A consultant makes a racist joke; staff laugh even if they know it is not right and should be addressed. Staff members treated differently due to religious observance e.g., observing sabbath, Friday prayer
Professional Values	1. What are the professional values expressed, and are some values omitted? 2. What should have happened according to professional expectations? 3. Were any values missing or compromised?	Alignment between actions and professional standards.	A patient is not treated with dignity due to workload pressures, despite policies promoting person-centred care. Staff dismiss concerns about inappropriate behaviour. Staff teaching on professional courses but not displaying or role-modelling this themselves.
Personal Values	1. How did my own beliefs or feelings influence my response? 2. Did I feel uncomfortable or conflicted? Why? 3. What would I do differently next time?	Self-awareness, emotions, implicit bias	A student avoids speaking up to avoid conflict, even though they recognise unfair treatment. A learner reflects afterwards that they wished they had acted differently.
Culture	1. What is considered “normal” in this setting? 2. How are people usually treated here? 3. Does this environment promote inclusion or exclusion?	Norms, routines, team behaviours	A ward routinely allocates less desirable tasks to certain staff or students' groups without questioning. “This is how things are done here” is used to justify behaviour in clinical practice. Junior academic staff unable to speak up or feel heard by senior leadership team- trying to raise concerns/Staff raising issues about toxic culture or environment
EDI education and awareness	1. Was I (or the team) prepared to respond to this situation? 2. Is EDI meaningfully embedded or superficially addressed? 3. What learning is needed to improve future practice?	Training, awareness, consistency between teaching and practice.	Staff complete mandatory online EDI training, but discriminatory behaviour is not challenged in practice. No space is provided to discuss or reflect on EDI issues during placement or clinical training or teaching sessions.

The revisions shown in Table 2 illustrate how student and educator feedback informed the evolution of the framework, transforming abstract domains into more accessible and actionable reflective prompts.

The revised prompts demonstrate how the Girandole can be operationalised as more than a conceptual framework. By translating its domains into practical reflective questions and context-specific examples, the framework becomes a flexible educational tool that can support teaching, learning, assessment, and professional development across multiple disciplines.

## 4 Potential applications across health professions education

The adapted Girandole framework has potential applications across health professions educationt (Table 3). Its six domains can support structured reflection withincontexts such as clinical and laboratory placements, professionalism teaching, portfolio-based learning and interprofessional education. These contexts are presented as illustrative possibilities rather than empirically evaluated applications. The framework may also offer educators a structured lens for curriculum review by prompting consideration of how power, hidden curricula, professional cultures and EDI are represented within formal and informal learning environments. Future implementation and evaluation will be required to establish its utility, acceptability and transferability across these settings.

**Table 3:** Illustrative potential applications of the Girandole framework across health professions education.

Educational context	Suggested Activity	Illustrative use
Clinical and Lab placements	Structured post-placement reflection	Students use Girandole-guided reflection to examine workplace hierarchies, lab culture, inclusion within teams, and experiences of inequities or inclusion.
Professionalism teaching	Analyse a vignette through the six vanes	Support discussion of ethical dilemmas, professional values, hidden curricula and inclusive practice across healthcare disciplines.
Portfolio-based modules	Reflective exercises	Guide for structured reflections on power, inclusion, belonging and social justice through students' professional development.
Interprofessional Education (IPE) activities	Compare disciplinary responses to a shared scenario	Facilitate dialogue between nursing, medicine, biomedical science and pharmacy students while recognising both discipline-specific perspectives and shared social justice challenges.

## 5 Discussion

The cross-disciplinary adaptation of the Girandole offers a structured approach to social justice reflection, helping to make visible power relationships, informal norms, and educational omissions.

The exploratory discussions here suggests that reflection cannot be separated from EDI literacy. Meaningful engagement with the Girandole requires learners to possess a baseline understanding of concepts such as equity, diversity, inclusion, and the hidden curriculum before they can critically recognise and interrogate what has been normalised, overlooked, or left untaught within their educational experiences. The workshop prompted reflection d that these concepts were not always immediately accessible to students, indicating a need for explicit educational scaffolding. Reflection alone cannot substitute for comprehensive EDI education; rather, it should complement and reinforce it. In this sense, the Girandole functions not only as a reflective tool but also as a diagnostic lens through which educators can examine the alignment between institutional EDI commitments and students' lived experiences.

Discussions of power, discrimination, and lived experiences can be emotionally challenging for both learners and educators. Reflective activities may surface experiences of bias, exclusion, or microaggressions that require sensitive facilitation and appropriate support. Educators therefore need the confidence and preparation to create psychologically safe learning environments and respond constructively to sensitive issues.

The Girandole should also be viewed as a complement to, rather than a replacement for, established reflective models such as Gibbs' Reflective Cycle and Johns' Model of Reflection. Its added value lies in explicitly foregrounding issues of equity, diversity, inclusion, and social justice that are often less visible within traditional reflective frameworks. Used alongside existing approaches, it can support a more socially informed form of reflection that considers broader structural influences on learning and professional development.

Finally, although the framework encourages critical inquiry and dialogue, it should not position learners or educators as solely responsible for addressing inequity. Many issues it seeks to illuminate are systemic in nature. Consequently, meaningful change requires organisational action, curriculum development, staff development, and sustained institutional commitment alongside individual reflection.

### 5.1 Future directions

The work reported here represents an early stage in the development of the Girandole as a cross-disciplinary reflective tool. Although the Girandole originated within nursing education, implementation discussions suggested that many of the issues represented by the framework transcend disciplinary boundaries. The examples identified relating not only to clinical nursing placements but also to pharmacy professionalism teaching, placement experiences, academic cultures, and biomedical science learning environments. This reinforces the potential value of a shared reflective language through which diverse health professions can engage.

The observations on the lack of EDI literacy across the students raise important questions for health professions education. How can EDI literacy be developed progressively throughout healthcare curricula? How can reflective approaches move beyond compliance-driven models towards deeper engagement with social justice? And how can institutions evaluate whether EDI teaching is genuinely transformative rather than tokenistic?

Future work will therefore focus on two interconnected areas:

1. Co-production with students and educators, empirical evaluation of the framework across multiple disciplines,
2. Development of funded interdisciplinary projects examining the contribution of the Girandole to reflective practice, EDI literacy, belonging, and social justice learning.

Rather than presenting a completed intervention, this paper represents an evolving programme of pedagogical scholarship aimed at strengthening the integration of EDI within health professions education. Further co-production and evaluation are required to establish its value across health professions curricula.

## Data Availability

The original contributions presented in the study are included in the article/Supplementary Material, further inquiries can be directed to the corresponding author.

## Footnotes

^1^The Girandole Framework (De Luca et al., 2026) named after the French word for a spinning pinwheel was developed through an interdisciplinary framework lab at a UK university. The Girandole maps six interconnected domains: power, hidden curriculum, professional values, personal values, culture, and EDI education and awareness. These domains/vanes collectively shape the EDI culture in clinical learning environments.
